# Interobserver variability of recall decisions between mammography readers in the English NHS breast screening programme: A comparison of interobserver variability measures

**DOI:** 10.1016/j.ejrad.2026.112723

**Published:** 2026-04

**Authors:** Laura Quinn, David Jenkinson, Sian Taylor-Phillips, Yemisi Takwoingi, Alice Sitch

**Affiliations:** a Department of Applied Health Sciences, University of Birmingham, Birmingham, UK; b NIHR Birmingham Biomedical Research Centre, University Hospitals Birmingham NHS Foundation Trust and University of Birmingham, UK; c School of Medicine, Keele University, Keele, UK; d Division of Health Sciences, Warwick Medical School, University of Warwick, Coventry, UK

**Keywords:** Breast cancer, Mammography, Observer variation

## Abstract

**Objectives:**

To evaluate interobserver variability between mammogram readers’ recall decisions in the English NHS breast screening programme, comparing different variability measures.

**Methods:**

Data from 401,682 women in 22 NHS centres who underwent mammographic screening interpreted independently by two mammogram readers were included. Percentage agreement, prevalence-adjusted bias-adjusted-kappa (PABAK), Gwet’s agreement coefficient (Gwet’s AC) and Cohen’s kappa were reported with 95% confidence intervals. Analyses were performed separately for women at first and subsequent screening appointments, by cancer diagnosis, reader recall rates and age group.

**Results:**

Of 86,287 women at first screening, 6,491 (7.5%) were recalled, compared to 9,488 (3.0%) of 315,395 at subsequent screenings. Percentage agreement, Gwet’s AC, and PABAK were lower for first screening than subsequent (93.6%, 95%CI: 93.4–93.7 vs 97.2%, 95%CI: 97.2–97.3), (92.3, 95%CI:92.1 to 92.5 vs 97.0, 95% CI: 97.0 to 97.1) and (87.2, 95%CI: 86.9–87.4 vs 94.4, 95%CI: 94.3–94.5), whereas Cohen’s kappa, which is biased downwards when prevalence of recall is lower, did not change (61.6, 95%CI: 60.7–62.5 vs 61.8, 95%CI: 61.0–62.5). Percentage agreement, Gwet’s AC, and PABAK were lower for women with cancer detected than without, but Cohen’s kappa showed the opposite pattern, driven by prevalence bias. Percentage agreement, Gwet’s AC, and PABAK were lower when one/both readers had high recall rates, but Cohen’s kappa showed no important pattern.

**Conclusions:**

Percentage agreement, Gwet’s AC, and PABAK showed lower agreement for interpreting the more challenging first screen, without assistance of previous mammograms, when women had cancer and when one/both readers had a high recall rate. Cohen’s kappa was heavily distorted by outcome prevalence. Despite widespread use, Cohen’s kappa is inappropriate for low prevalence settings such as screening, or making comparisons when prevalence varies.

## 1 Introduction

Breast cancer screening uses mammograms to detect cancer in asymptomatic women, which are examined by one or two readers. Interobserver variability is the variability between two independent readers when deciding whether to recall a woman for further testing due to the suspicion of breast cancer. It is a critical measure for ensuring delivery of consistent care, and when considering the use of new technology in screening, to assess whether radiologists can consistently interpret the new technology/images, and to understand the potential impact of artificial intelligence which does not contain the variability of radiologists.

In breast cancer screening, variability refers to the differences in decisions made by different observers, while agreement refers to the similarity or consistency between observers’ decisions. Variability measures are single values used to estimate interobserver variability, there are different types of measures depending on the type of outcome. For binary outcomes, such as whether or not to recall a woman for further testing, measures include percentage agreement, Cohen’s kappa, Prevalence-adjusted-bias-adjusted-kappa (PABAK) and Gwet’s agreement coefficient (Gwet’s AC). Apart from percentage agreement, which just measures the observer agreement between observers, the other variability measures account for chance agreement, which is the agreement between readers if it occurred by random chance. A systematic review of imaging studies examining interobserver variability in 2019 found that Cohen’s kappa was the most commonly used for categorical outcomes [1], which aligns with previous research [2]. There is some literature available showing that Cohen’s kappa does not perform well when the prevalence is very low or high [3], [4]. As this study focuses on screening for breast cancer and the prevalence is expected to be very low, we will look at the effect of low prevalence on different variability measures.

Previous studies have evaluated the interobserver variability of breast cancer diagnoses, primarily looking at breast density and breast imaging-reporting and data system (BI-RADS) [5], [6], [7], [8], [9] in hospital settings using weighted or Cohen’s kappa, except one study using Gwet’s AC [10]. No previous studies have compared interobserver variability using different variability measures on breast screening readers. A study in the Netherlands reported cancer referral and detection rates, but no variability measures were reported and readers were not always blinded to other interpretations [11]. In Ireland, two studies reported consensus reviews for discordant recall decisions [12], [13].

Interobserver variability for recall rates may be affected by several factors. Most notably recall rate varies by readers and between countries. Having a low recall rate can lead to missed cancer cases, however, having a high recall rate may only lead to a small increase in detected cancer cases and unnecessary tests for women without cancer [14]. The NHS breast screening programme sets an acceptable and achievable recall rate for first screenings and subsequent screenings. The screening programme quality assurance guidelines recommend pairing inexperienced readers with experienced readers, and also pairing readers with low recall rates with readers with high recall rates to balance each other out although operationalising this in a busy breast screening unit is challenging [15], [16], [17]. The Dutch screening programme has one of the lowest recall rates [14] while the USA has a much higher recall rate compared to other countries [18], [19]. Whether specific reader recall rates affect interobserver variability between mammogram readers is unknown. Other factors that may be linked to interobserver variability are age of women undergoing screening and whether a woman ultimately had a cancer diagnosis. Breast density decreases as women get older [20] which could make mammograms easier to interpret. It is expected that agreement between observers would increase for an older age group, however, to our knowledge, there are no existing studies reporting interobserver variability by age group. In this study, breast cancer diagnoses could be made at the initial screening, during the interval before the next screening, or at the next screening. All available screening and follow-up information was used to establish whether or not a woman has breast cancer. However, it remains unknown whether interobserver variability is different between women with and without a breast cancer diagnosis.

This study aims to estimate the interobserver variability of recall decisions between mammography readers in the NHS breast cancer screening program for first and subsequent screening appointments, exploring the effect of low prevalence in breast cancer screening on different variability estimates. In addition, factors that could influence interobserver variability such as cancer diagnosis, reader recall rates and age groups, were explored.

## 2 Methods

### 2.1 Study design and population

The data in this study is from the CO-OPs trial, a pragmatic, multicentre, two-arm, double-blind, cluster-randomised controlled trial. All of the data in this study has been used in a previous study [21] which looked at the effect of double readers by a second reader, whereas in this study we have calculated the interobserver variability between readers and only included patients where readers were blinded to each other’s interpretations of patients’ mammograms.

Institutional Review Board approval was given by the Coventry and Warwickshire NHS Research Ethics Committee on June 27, 2012 (WM/0182) for the CO-OPs trial, which included approval for further research. Written informed consent for the CO-OPs trial was obtained from each director of breast screening for the CO-OPS Trial. All patient and reader details were de-identified before sending to the researchers. The CO-OPs trial included women undergoing screening mammography between December 2012 and November 2014 at 46 NHS breast screening centres in England. Each centre collected data for one year. For the CO-OPs trial, the eligibility criteria were:

• Centres were considered eligible if screening mammograms were interpreted by two readers and the centre had at least one piece of digital mammogram equipment
• All women who underwent screening mammography at the eligible centres were included
• All readers in the trial were required to examine a minimum of 5000 mammograms per year [21].

Further eligibility criteria for this interobserver variability study were:

• Women must have had their digital mammograms interpreted independently by two readers to decide whether to recall for further investigation on the suspicion of breast cancer.
• Women were excluded if the second reader reported being unblinded to the first reader’s interpretation or if an error occurred where only one reader interpreted the mammogram.

Note, as the interobserver study uses a subset of the CO-OPs trial data, readers will have examined fewer than the minimum number (5000) of mammograms per year.

### 2.2 Statistical analysis

Descriptive characteristics of the women who underwent screening mammograms, the readers, recall rates and cancer detection rates were reported using frequencies and percentages, means and standard deviations or medians and interquartile ranges, as appropriate.

Interobserver variability of recall decisions between mammogram readers was estimated for first and subsequent screening appointments separately using percentage agreement, prevalence-adjusted-bias-adjusted kappa (PABAK), Gwet’s agreement coefficient (Gwet’s AC) and Cohen’s kappa. Gwet’s AC was included because it is less affected by prevalence and marginal distribution than Cohen’s kappa, whereas PABAK adjusts for both prevalence and bias, providing complementary insights in this low-prevalence screening setting. Although PABAK and Gwet’s AC both address limitations of Cohen’s kappa, they differ in how chance agreement is calculated and therefore may yield different numerical values. Further descriptions of the variability measures are given in Table S1. All variability measures were reported with 95% confidence intervals.

Additionally, interobserver variability measures were calculated by breast cancer diagnosis and by the level of reader recall rate for first and subsequent screening appointments separately. Analysis was also completed by age group and a combination of age group and breast cancer diagnosis for subsequent screening appointments only.

Breast cancer diagnosis was defined when a woman had a cancer detected at the current screen, in-between the current screen and the next planned screen (interval cancer), or at the following scheduled screen.

Reader recall rates were categorised into low, medium, and high for each reader based on the first six months of data from their centre and the standards for first and subsequent screening set by the NHS breast screening programme [Table 1]. Reader recall rates were rounded to the nearest percentage point. To calculate reader recall rates, a minimum of 250 interpretations per reader in the first six months for first and subsequent screening appointments was required.

**Table 1:** Levels of reader recall rates.

Level of reader recall rate	First screening	Subsequent screening
Low	≤6%	≤4%
Medium	7% to 9%	5% to 6%
High	≥10%	≥7%

Age was initially categorised into women aged less than 60 years and women aged 60 years or older for subsequent screening, a pragmatic choice based on the midpoint age for the standard screening programme. Additionally, we investigated further age groups with women split into 3-year categories, as this is the standard interval between screens. Age group was not used as a subgroup for first screening appointments, as the majority of women will undergo their first screen when they are 50 years old.

## 3 Results

### 3.1 Participant and reader characteristics

**Fig. 1:**
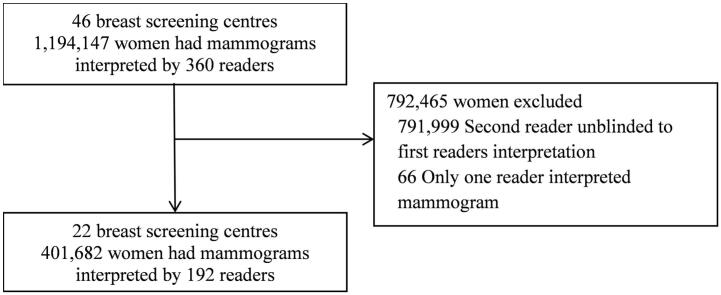
Flow diagram.

In this study, there were 401,682 women from across 22 centres who had their mammograms interpreted independently by two different readers (total number of readers was 192) [Fig. 1]. Of the 401,682 women, 86,287 (21.48%) underwent first screening appointments and 315,395 (78.52%) underwent subsequent screening appointments between December 2012 and November 2014. Due to the length of the study period, each woman only underwent one screening mammograms.

Most women undergoing first screening were less than 60 years old (97.13%), while under half of the women undergoing subsequent screening were less than 60 years old (40.83%). Of the 192 mammogram readers, 186 interpreted first screenings and the median number of interpretations per reader was 1,000 (IQR: 221 to 1,453). For subsequent screening, 189 readers interpreted mammograms and the median number of reads was 3,565 (IQR: 735 to 4,834).

### 3.2 Recall and cancer detection rates

The first reader to interpret each image recalled 8,255 (9.57%) women with first screening and 12,308 women (3.90%) with subsequent screening for further investigation [Table 2]. Recall percentages for the second reader were similar (8.86% for first and 3.61% for subsequent screening). A final decision to recall (decided by arbitration if readers disagreed) was made for 7.52% of first screens and 3.01% of subsequent screens.

**Table 2:** Recall and cancer detection rates. Frequency and percentages reported.

	First screen (N = 86,287)	Subsequent (N = 315,395)
Recall rate		
First reader	8,255 (9.57)	12,308 (3.90)
Second reader	7,645 (8.86)	11,388 (3.61)
Final decision	6,491 (7.52)	9,488 (3.01)
Initial screening		
Cancer detected	720 (0.83)	2,819 (0.89)
Between initial and next screen		
Cancer detected (interval)	172 (0.20)	641 (0.20)
Next screening		
Cancer detected	387 (0.45)	2,117 (0.67)
No cancer detected	80,367 (93.14)	275,478 (87.34)
No data available	4,641 (5.38)	34,340 (10.89)

Similar percentages of cancer were detected at the initial screen (0.83% vs 0.89%), between the initial and next screen (interval cancer, 0.20% vs 0.20%) and at the next screen (0.45% vs 0.67%) for first and subsequent screening appointments.

### 3.3 Interobserver variability

For 86,287 women attending their first screening appointment, the percentage agreement between readers for recall decisions was 93.58% (95% CI: 93.41% to 93.74%), meaning for 80,745 women, both readers agreed on whether to recall for further investigation [Table 3]. For first screening appointments, Gwet’s AC was 92.29 (95% CI: 92.08 to 92.50), PABAK was 87.16 (95% CI: 86.93 to 87.38) and Cohen’s kappa was 61.61 (95% CI: 60.69 to 62.54). Percentage agreement of 93.58% and Gwet’s AC of 92.29 represent very high agreement (adjusted for chance agreement). A PABAK value of 87.16 represents almost perfect agreement (adjusted for chance agreement, prevalence and bias) and a Cohen’s kappa value of 61.61 represents substantial agreement (adjusted for chance agreement only) according to Landis and Koch’s interpretation [22].

**Table 3:** Contingency tables and variability estimates of readers recall decisions for first and subsequent screening appointments separately.

First screening					Subsequent screening				
		R2 decision					R2 decision		
		Recall	Do not recall	Total			Recall	Do not recall	Total
R1 decision	Recall	5,179	3,076	8,255	R1 decision	Recall	7,488	4,820	12,308
	Do not recall	2,466	75,566	78,032		Do not recall	3,900	299,187	303,087
	Total	78,642	7,645	86,287		Total	11,388	304,007	315,395

Percentage agreement (95% CI)	93.58 (93.41 to 93.74)	Percentage agreement (95% CI)	97.24 (97.18 to 97.29)
Gwet’s AC (95% CI)	92.29 (92.08 to 92.50)	Gwet’s **AC (95% CI)**	97.02 (96.96 to 97.08)
PABAK (95% CI)	87.16 (86.93 to 87.38)	PABAK (95% CI)	94.48 (94.40 to 94.56)
Cohen’s kappa (95% CI)	61.61 (60.69 to 62.54)	Cohen’s kappa (95% CI)	61.77 (61.03 to 62.51)

For the 315,395 who had previously attended screening, the percentage agreement between readers for recall decisions was 97.24% (95% CI: 97.18% to 97.29%), Gwet’s AC was 97.02 (95% CI: 96.96 to 97.08), PABAK was 94.48 (95% CI: 94.40 to 94.56) and Cohen’s kappa (61.77, 95% CI:61.03 to 62.51). Percentage agreement, Gwet’s AC, and PABAK were higher for subsequent screening compared to first screening as would be expected due to the availability of previous mammograms, while Cohen’s kappa values were very similar.

*Impact of cancer diagnosis, reader recall rates and age groups*.

**Fig. 2:**
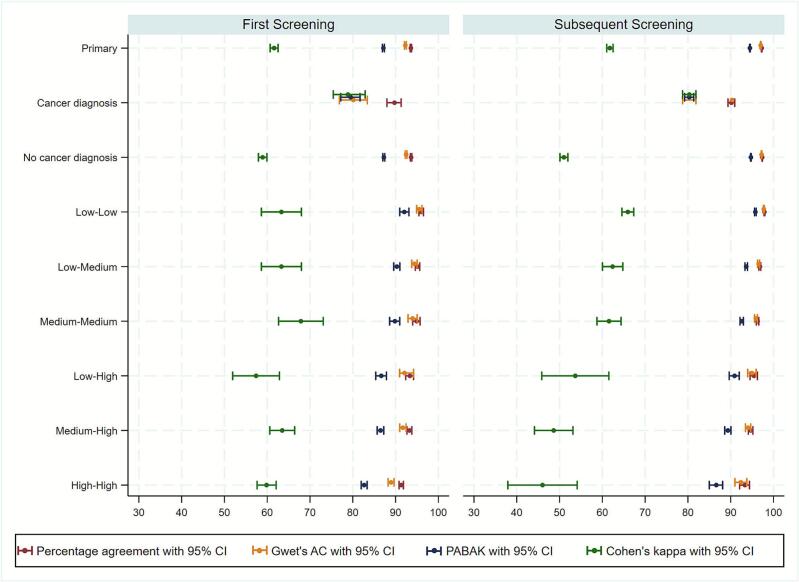
Interobserver variability for recall decisions between mammogram readers for all women, cancer diagnosis and reader recall groups. Variability estimates and 95% confidence intervals are shown by first and subsequent screening appointments.

For all women, percentage agreement, Gwet’s AC, and PABAK values for recall decisions between mammogram readers were lower for women who ultimately had a cancer diagnosis compared to those without a cancer diagnosis during the screening process [Table 4*,* Fig. 2]. This is to be expected because the vast majority of women without cancer are easily classified as not requiring recall by most mammography readers. However, Cohen’s kappa values followed the opposite pattern, with higher values for women with cancer compared to those without, regardless of whether they were first screens (78.87, 95% CI: 75.44 to 82.29 vs 58.94, 95% CI:57.95 to 59.93) or subsequent screens (80.32, 95% CI:78.75 to 81.88 vs 51.02, 95% CI:50.09 to 51.94). This is likely due to the prevalence bias of Cohen’s kappa, in which the measure systematically biases towards a higher kappa when prevalence is not very low or high.

**Table 4:** Contingency tables and variability estimates of readers recall decisions by cancer diagnosis for first and subsequent screening appointments separately.

First screening with cancer diagnosis					Subsequent screening with cancer diagnosis				
		R2 decision					R2 decision		
		Recall	Do not recall	Total			Recall	Do not recall	Total
R1 decision	Recall	687	51	738	R1 decision	Recall	2,564	211	2,775
	Do not recall	80	461	541		Do not recall	338	2,464	2,802
	Total	767	512	1,279		Total	2,902	2,675	5,577

Percentage agreement (95% CI)	89.76 (88.09 to 91.42)	Percentage agreement (95% CI)	90.16 (89.37 to 90.94)
Gwet’s AC (95% CI)	80.14 (76.87 to 83.40)	Gwet’s **AC (95% CI)**	80.32 (78.75 to 81.88)
PABAK (95% CI)	79.52 (77.20 to 81.70)	PABAK (95% CI)	80.32 (79.24 to 81.35)
Cohen’s kappa (95% CI)	78.87 (75.44 to 82.29)	Cohen’s kappa (95% CI)	80.32 (78.75 to 81.88)

First screening without cancer diagnosis					Subsequent screening without cancer diagnosis				
		R2 decision					R2 decision		
		Recall	Do not recall	Total			Recall	Do not recall	Total
R1 decision	Recall	4,492	3,025	7,517	R1 decision	Recall	4,492	4,609	9,533
	Do not recall	2,386	75,105	77,491		Do not recall	3,562	296,723	300,285
	Total	6,878	78,130	85,008		Total	8,486	301,332	309,818

Percentage agreement (95% CI)	93.63 (93.47 to 93.80)	Percentage agreement (95% CI)	97.36 (97.31 to 97.42)
Gwet’s AC (95% CI)	92.47 (92.26 to 92.67)	Gwet’s **AC (95% CI)**	97.21 (97.15 to 97.27)
PABAK (95% CI)	87.26 (87.03 to 87.48)	PABAK (95% CI)	94.72 (94.64 to 94.80)
Cohen’s kappa (95% CI)	58.94 (57.95 to 59.93)	Cohen’s kappa (95% CI)	51.02 (50.09 to 51.94)

Reader recall rate was calculated for the 144 readers who interpreted at least 250 mammograms in the first six months their centre participated in the study. These recall rates were used to categorise the readers for the remaining six months into low, medium and high recall rates separately for first and subsequent screenings. For the analysis by reader recall groups, 185,733 women were included and 134 readers (10 readers did not interpret mammograms in the last year of study). For first and subsequent screens, percentage agreement, Gwet’s AC, and PABAK were highest between readers both classified as having low recall rates and lowest when both readers were classified as having high recall rates [Table S2*,* Fig. 2]. When there was at least one high recall reader, percentage agreement, Gwet’s AC, and PABAK were lower. In this setting, where fewer than one third of women recalled have cancer, most extra recalls from high recall readers do not have cancer, and different high recall readers appear to be recalling different additional women driving the higher disagreement. Cohen’s kappa did not show any significant pattern in agreement between the reader recall rate pairings, with relatively wide confidence intervals compared to the other measures. Across all combinations of reader recall groups, percentage agreement, Gwet’s AC, and PABAK were higher for subsequent compared to first screening [*see contingency tables in* Table S4].

**Fig. 3:**
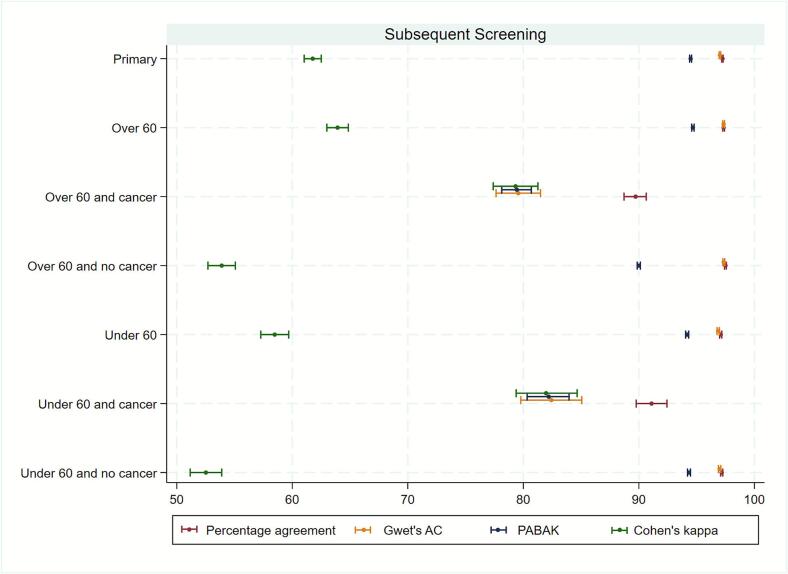
Interobserver variability for recall decisions between mammogram readers for all women, by age group, and cancer diagnosis. Variability estimates and 95% confidence intervals are shown for subsequent screening only.

For subsequent screening, percentage agreement, Gwet’s AC, and PABAK were slightly higher for all women over 60 compared to those under 60 [Fig. 3, *see* Table S3 *for summary and* Table S5 *for contingency tables*]; higher for women over 60 compared to under 60 for women without a cancer diagnosis up to or at her next screen and similar across age groups in women who had a cancer diagnosis up to or at her next screen. Cohen’s kappa showed the same pattern of increasing agreement with women’s age, as older women have more fatty tissue which is transparent on mammograms and easier to interpret which may increase agreement. When evaluating 3-year age group percentage agreement, PABAK and Cohen’s kappa were similar across age groups overall, and by cancer diagnosis [see Table S3].

## 4 Discussion

Over 400,000 women underwent screening mammograms which were interpreted independently by two readers to decide whether or not to recall for further investigation due to the suspicion of breast cancer. Percentage agreement, Gwet’s AC, and PABAK were higher for subsequent screening compared to the first screening which is expected as previous screening mammograms can aid readers with their decisions [23]. However, Cohen’s kappa values were similar, with overlapping confidence intervals. Percentage agreement, Gwet’s AC, and PABAK were higher for women who did not have a cancer diagnosis compared to those who did, driven by the large numbers of women without cancer in whom most readers would easily agree that there are no features which merit recall. In contrast, Cohen’s kappa was higher for women with a cancer diagnosis than for those without one, because the pattern of greater disagreement in women with cancer was masked by the prevalence biases of Cohen’s kappa. The numerical differences between Gwet’s AC and PABAK reflect differences in how chance agreement is calculated; however, both measures are less influenced by prevalence than Cohen’s kappa and show consistent patterns of agreement. Percentage agreement, Gwet’s AC, and PABAK were lower when one or both readers had a high recall rate. It appears that high recall readers are recalling different additional women driving the higher disagreement, which makes sense in a context where the majority of women recalled do not have cancer. Cohen’s kappa had wider confidence intervals and showed no significant pattern of agreement by reader recall rate. This example demonstrates that Cohen’s kappa whilst the most commonly used variability measure in radiology, has a prevalence bias so gives erroneous conclusions when prevalence varies, and interpretation must take account of the prevalence in the study population. Using Landis and Koch’s arbitrary interpretations, a conclusion of ‘substantial agreement’ could be erroneously made when there is relatively low agreement if the prevalence in the dataset is not very low or high.

Our findings have several clinical implications for breast screening practice. Lower agreement between readers for first screens highlights the challenge of interpreting mammograms without prior images for comparison, suggesting that additional support or training for readers may help to improve consistency. Previous studies concur that access to previous mammograms improves readers ability to interpret images more consistently and accurately [24], The lower agreement when one or both readers had high recall rates indicates that higher recall rates contribute to interobserver variability and may need to be addressed through reader pairings, as already recommended in quality assurance guidelines [17]. Lastly, lower agreement among women with cancer diagnoses underlines the interpretative challenges of subtle signs of cancer and highlights the critical role of arbitration and consensus review in resolving discordant cases to support accurate detection.

Our study is one of the few to focus on interobserver variability between mammogram readers for recall decisions in a large screening programme, most focus on breast density and BI-RADS in hospital-based studies. Of studies looking at screening mammograms, most studies focus on the discordance of recall decisions and only percentage agreement was reported [12], [13], [25]. A study in Ireland that looked at discordant recall decisions for over 120,000 mammograms reported 99% agreement which is higher than this study, however, there were only seven readers from two centres, women could have multiple mammograms during the study and screen-film mammograms were used [12]. Subsequently, another study by the same authors used digital mammograms with 98.6% agreement, however other differences with our study such as a small sample of readers and multiple mammograms were still present. One study in Korea reported 88.7% agreement and a kappa value of 0.27 which is lower compared to this study [26], however, the study only included 1000 mammograms with 12 cancer cases and collapsed a seven-category measurement into whether or not to recall. To our knowledge no previous studies have compared different variability measures for breast screening radiologists.

The main strength of this study is the population of the study, a large sample of women who underwent screening mammograms and readers who interpret mammograms for recall decisions for the NHS breast screening programme in England were included. Some potential limitations of this study include the blinding of readers, all readers in this study reported being blind to other readers interpretations, however, it is possible that some readers looked which could cause confirmation bias. Also, due to the way reader logins were created, it is possible that one ID may have been used for two readers with the same name in different centres. We believe this happens infrequently and is purely at random. Reader recall rate classifications for the subgroup analysis were based on the first six months of data. We were unable to verify whether these classifications remained stable throughout the study period; however, substantial changes are unlikely given the high volume of annual readings required. Data were collected between 2012 and 2014, when digital mammography systems met quality standards at the time. All digital mammography systems are subject to rigorous and regular standardised quality assurance testing and must meet specification standards [27]. Although there have been some advances in digital mammography imaging technologies since then, digital mammography remains a core screening method, with changes reflecting small, incremental improvements rather than major changes. Therefore, the core patterns of interobserver variability and comparative performance of variability measures are expected to remain relevant.

With Artificial Intelligence (AI) implementation in breast screening being explored with the aim of reducing variability and workload while increasing accuracy, current evidence remains mixed regarding its performance and how AI integrates into reader workflow and decision-making [28], [29]. This study found interobserver variability among human readers, highlighting the complexity of integrating AI systems. AI systems typically allow users to choose a threshold for recall decisions, and this choice influences AI performance. Importantly, selecting an appropriate AI threshold must consider the variability of the human readers with whom AI is intended to be integrated. Despite the promise of AI, understanding baseline interobserver variability between human readers remains essential as a reference point for safely evaluating and integrating AI into the breast screening programme. Future evaluations of AI-assisted breast screening should benchmark performance against established patterns of human interobserver variability.

In conclusion, the percentage agreement, Gwet’s AC, and PABAK values between mammogram readers for recall decisions in the NHS breast cancer screening programme were lower for interpreting the more challenging first screen, without assistance of previous mammograms for comparison, for women who ultimately had a breast cancer diagnosis and when one or both of the readers had high recall rates. Cohen’s kappa were affected by the prevalence of the outcome, and despite widespread use is not appropriate for measuring agreement in a low prevalence setting such as screening, or making comparisons when prevalence varies. Percentage agreement, Gwet’s AC, and PABAK are less affected by prevalence and should be used instead.

Funding

This study was funded by a National Institute for Health Research (NIHR) Doctoral Research Fellowship (DRF), reference number NIHR300606. LQ is supported by the NIHR Applied Research Collaboration (ARC) West Midlands. STY is funded by an NIHR Research Professorship (NIHR302434). The funding sources had no role in the collection, analysis, or interpretation of the data, or in the decision to submit the manuscript for publication.

Ethics statement

Institutional Review Board approval for the original trial was obtained from Coventry and Warwickshire National Health Service [NHS] Research Ethics Committee, June 27, 2012, WM/0182.

## CRediT authorship contribution statement

**Laura Quinn:** Writing – review & editing, Writing – original draft, Methodology, Formal analysis, Data curation, Conceptualization. **David Jenkinson:** Writing – review & editing, Data curation, Conceptualization. **Sian Taylor-Phillips:** Writing – review & editing, Supervision, Methodology, Data curation, Conceptualization. **Yemisi Takwoingi:** Writing – review & editing, Supervision. **Alice Sitch:** Writing – review & editing, Supervision, Methodology, Conceptualization.

## Declaration of competing interest

The authors declare that they have no known competing financial interests or personal relationships that could have appeared to influence the work reported in this paper.
